# A Synergistic Formulation of Plant Extracts Decreases Postprandial Glucose and Insulin Peaks: Results from Two Randomized, Controlled, Cross-Over Studies Using Real-World Meals

**DOI:** 10.3390/nu10080956

**Published:** 2018-07-25

**Authors:** Edyta Adamska-Patruno, Katarzyna Billing-Marczak, Marek Orlowski, Maria Gorska, Marcin Krotkiewski, Adam Kretowski

**Affiliations:** 1 Clinical Research Centre, Medical University of Bialystok, M.C. Sklodowskiej 24A, 15-276 Bialystok, Poland; adamkretowski@wp.pl; 2 Marmar Investments Sp. z o.o., Slominskiego 15, lok 509, 00-195 Warsaw, Poland; kmarczak@marmarinvestment.pl (K.B.-M.); morlowski@adiuvoinvestments.com (M.O.); 3 Department of Endocrinology, Diabetology, and Internal Medicine, Medical University of Bialystok, M.C. Sklodowskiej 24A, 15-276 Bialystok, Poland; mgorska25@wp.pl; 4 Department of Neurological Rehabilitation, Gothenburg University Hospital, SE-405 30 Gothenburg, Sweden

**Keywords:** plant extracts, natural product, postprandial glucose peaks, postprandial insulin peaks, pre-diabetes, metabolic syndrome, type 2 diabetes, overweight, obesity

## Abstract

This study investigated the efficacy of a plant-derived dietary supplement with respect to decreasing postprandial glucose and insulin peaks after the intake of real-world meals. Two randomized, double-blind, placebo-controlled, cross-over experiments were conducted on healthy subjects who received a supplement containing extracts of white mulberry, white bean, and green coffee or one containing the three extracts with added fibre before consuming high-GI/GL (glycaemic index/glycaemic load) meals. In study one, 32 subjects received an investigational product/placebo before a standardized meal at two visits. In study two, 150 subjects received an investigational product/placebo before five different standardized meals. Postprandial glucose and insulin concentrations were lower 20–35 min after meal intake among subjects taking the investigational product, and fewer episodes of postprandial reactive hypoglycaemia were noted. For example, after consuming breakfast cereal with milk, lower glucose peaks were observed for the investigational product (vs. placebo) after 20 min (100.2 ± 1.97 vs. 112.5 ± 3.12 mg/dL, respectively; *p* < 0.01); lower insulin peaks were noted at the same time point (45.9 ± 4.02 IU/mL vs. 68.2 ± 5.53 IU/mL, respectively, *p* < 0.01). The combined formulation decreases the adverse consequences of high-GI/GL meal consumption. It can be an effective dietary supplement for the management of metabolic syndrome and type 2 diabetes mellitus.

## 1. Introduction

Obesity is an increasingly important clinical problem [1]. This multifactorial disorder is a major risk factor for type 2 diabetes mellitus (T2DM), hypertension, cardiovascular events, and many other diseases that can lead to morbidity and mortality [2]. Increasing rates of obesity [1] and T2DM threaten both our health and our health care system, primarily due to the relative dearth of effective therapies.

Currently, successful prevention and treatment of obesity consist of various dietary strategies, which can be more or less effective for different types of patients [3]. One of the dietary approaches is the low-glycaemic index (GI) and low-glycaemic load (GL) diet; this diet is based on consuming carbohydrate-containing foods that cause the smallest change in blood glucose and insulin levels following meal consumption. Many studies have shown positive associations for GI and GL with risk factors for metabolic diseases, and there is increasing evidence that low-GI and low-GL dietary patterns can prevent disorders such as overweight, obesity, glucose intolerance, pre-diabetes, diabetes, and cardiovascular disease, among others [4]. However, one of the biggest barriers to long-term maintenance of weight loss is poor adherence to dietary restrictions [3]. Therefore, developing ways to improve dietary adherence and supporting those unable to follow dietary restrictions are of utmost importance for the prevention and treatment of metabolic disease.

The main effect of high-GI and high-GL food intake are high blood glucose concentration and its metabolic consequences, such as high insulin levels, reactive hypoglycaemia, as well as its prodromal symptoms like sudden and intense hunger leading to cravings and overeating [5]. Therefore, decreasing carbohydrate digestion and absorption may reduce the impact of carbohydrate intake on blood glucose levels. Indeed, α-glucosidase inhibitors like acarbose, which prevent carbohydrate digestion by lining the intestine with cells, have already been used in the treatment and prevention of T2DM [6,7]. Acarbose treatment was also associated with a significant reduction of cardiovascular risk in high-risk populations [8,9]. Despite these beneficial effects, acarbose is associated with a number of gastrointestinal side effects including flatulence and diarrhoea [7].

Some plant extracts also contain bioactive ingredients that may inhibit digestion and reduce carbohydrate absorption; in particular, the leaves of some genotypes of mulberry (*Morus alba* L.) contain a number of bioactive phytochemicals including flavonoids, steroids, triterpenes, quercetin, kaempferol, chlorogenic acids, and caffeic acid, as well as the α-glucosidase inhibitor 1-deoxynojirimycin (DNJ) [10,11]. As such, mulberry leaves have the potential to exhibit a number of anti-diabetic effects, including inhibition of α-glucosidase, sucrase, and maltase enzyme activity, thereby reducing carbohydrate metabolism and lowering blood glucose levels [12]. In addition, the common white bean (*Phaseolus vulgaris*) produces an α-amylase inhibitor that prevents starch digestion; the white bean extract was found to reduce the postprandial plasma glucose and to eliminate the subsequent fall in glucose levels [13]. Furthermore, chlorogenic acid, found in green coffee, can reduce glucose uptake in the intestine by dissipating the Na^+^ electrochemical gradient that drives glucose accumulation [14]. Chlorogenic acid may also inhibit the activity of hepatic glucose-6-phosphatase, which is implicated in glucose homeostasis [15]. Therefore, we developed an innovative dietary supplement containing the abovementioned plant-derived extracts, which may help minimalize the negative consequences of consuming high-GI and high-GL meals.

We hypothesized that a combination of white mulberry, white bean, and green coffee extracts (which contain the abovementioned bioactive ingredients) might have a synergistic/additive effect on glucose metabolism with fewer side effects than acarbose. This combined dietary supplement may be particularly beneficial for patients with problems adhering to dietary restrictions. In these studies, we investigate the efficacy and safety of the combined plant-derived dietary supplement depending on different dietary meal intakes in the real world.

## 2. Materials and Methods 

### 2.1. Study Design

**Figure 1:**
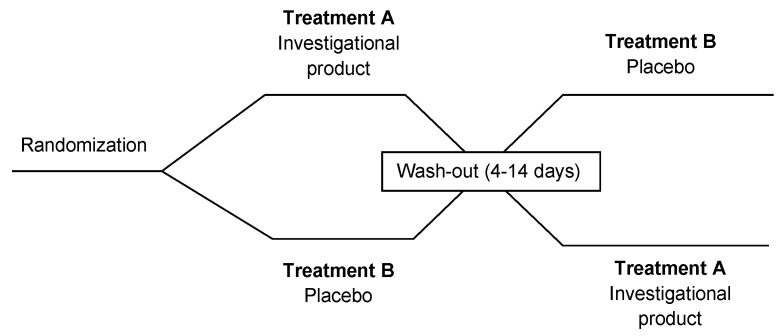
Double-blind, randomized, placebo-controlled, cross-over study design.

Two single-centre, randomized, double-blind, placebo-controlled, cross-over studies were performed (Figure 1). Study 1 investigated the effects of two dietary supplements (investigational product (IP)-A and IP-B) on postprandial glycaemia and insulin levels in healthy subjects (compared to placebo) after a high-GI/GL meal. Study 2 investigated the effects of IP-A on postprandial glycaemia and insulin levels in healthy subjects after five different meals (i.e., all high-GI/GL meals but at various levels).

### 2.2. Investigational Products

In Study 1, two products were investigated: IP-A (a complex of 600 mg white mulberry, 1200 mg white bean extract, and 400 mg green coffee) and IP-B (a complex of 600 mg white mulberry, 1200 mg white bean extract, 400 mg green coffee, with the addition of 2000 mg inulin and 3000 mg glucomannan). In Study 2, only IP-A was investigated. 

In both products, white mulberry extract contained 3.12% of DNJ (standardization for minimum 3%), the white bean extract was standardized to contain a minimum 4000 units of α-amylase inhibitor, and the green coffee extract contained 52.3% of chlorogenic acid (standardization for minimum 50%) and 1.6% of caffeine (standardization for maximum 2%). Additionally, IP-B contained 95% purity inulin and 90% purity glucomannan. 

The IPs and placebo for each study were prepared by Ichem Sp. z o.o., Lodz, Poland, in such a manner that they could not be distinguished based on their appearance, weight, flavour, or volume. The IPs and placebo were prepared as powders, dissolved in 200 mL of boiled water, and administered as oral suspensions at room temperature.

### 2.3. Inclusion and Exclusion Criteria

Healthy male and female participants (aged 18–64 years) with body mass index (BMI) ranging from 22.99 to 29.99 kg/m^2^ were included in the studies (Table 1). Glucose metabolism disorders, endocrine disorders, renal, liver and digestive system diseases, gastroenterological and bariatric surgeries or procedures, and any other diseases that could influence the study results constituted exclusion criteria. People who received pharmacological treatment or used any other products with documented or unknown influences on glucose metabolism were also excluded from the study. Women were allowed to take hormonal agents (contraceptives or hormone replacement therapy).

### 2.4. Meals

In Study 1, subjects received a white bread roll as a standardized meal. In Study 2, subjects received one of the following five meals: a coke-type beverage and puffcorn; breakfast cereal with vanilla milk; raspberry muffin and liquid blueberry yoghurt; French fries with ketchup; or cheese pasta. The nutritional information (kcal, total carbohydrate, protein, and fat content) for the high-GI/high-GL meals tested in each study is presented in Table 2.

### 2.5. Study Procedures

Subjects who met the selection criteria were randomized and received the IPs/placebo according to the randomization table (using randomly chosen permutations). Subjects were instructed to maintain their regular lifestyle throughout the study and to avoid coffee, alcohol, and excessive physical exercise for three days prior to the test. After overnight fasting (for at least 11 h), the subjects arrived at the laboratory on the test day (at the same time for every study visit). Fasting blood was collected, and subjects received the IP/placebo. A single dose of IP/placebo was administered orally 15 min before the start of meal consumption. Blood samples were collected to determine blood glucose and insulin concentrations before IP/placebo administration (0 min) and 20, 35, 50, 65, 80, 95, and 125 min after meal intake.

### 2.6. Anthropometric Measurements

Weight (InBody 720, Biospace, Seoul, Korea) and height (stadiometer Seca 264, Seca GmbH & Co. KG, Hamburg, Germany) were measured by trained researchers in a standardized way, and Body Mass Index (BMI) was calculated based on weight divided by height squared.

### 2.7. Biochemical Measurements

Serum glucose concentration was measured by colorimetric methods using commercially available test kits (Roche Diagnostics, Rotkreuz, Switzerland) and a Cobas c111 analyzer (Roche Diagnostics, Rotkreuz, Switzerland). Serum insulin concentration was estimated by an immunoradiometric assay (IRMA) using commercially available test kits (DiaSource, Louvain-la-Neuve, Belgium), and assay tubes were counted in a Wallac Wizard 1470 Automatic Gamma Counter (Perkin Elmer, Life Science, Turku, Finland). All measurements were repeated twice.

### 2.8. Statistical Analysis

The estimation of group size was conducted on the basis of the results of the animal study: “Implementation report for task II–Investigation into the effect of individual extracts on the postprandial glucose value in animals” within the project “Development of an innovative dietary supplement capable of decreasing the glycaemic index of consumed meals.” AUC (areas under the curves) calculated in the above study was used as an analyzed variable. The following targets were assumed:

- Independent samples Student’s *t*-test will be conducted.
- Hypothesis H0: means in both populations are equal, therefore m1 = m2.
- Hypothesis H1: means in both populations are different, therefore m1 ≠ m2.
- Significance level (probability of type I error): a = 0.05.
- Standard deviation in the population: s = 60 and s = 75.
- Target power: 80% and 90%.

Student’s *t*-test is the most popular method for evaluating differences between means in two groups. If the above-mentioned conditions (normal distribution) are not met, a relevant non-parametric test can be conducted (Mann-Whitney U test). The analysis was conducted with the tool “Test power analysis and determination of group sizes for experiment planning–Calculation of required sample size” in the STATISTICA 10.0 package.

With the weakest conditions (a = 0.05; s = 60, and target power at 80%) and assuming an examination of composition I, II, and III for sucrose tolerance test, the following minimum size was obtained: 4.0 for each composition. With the soluble starch tolerance test, the obtained sizes for composition I, II, and III were, respectively: 7.0, 9.0, and 6.0. With the strongest conditions (a = 0.05; s = 75, and target power at 90%) the sizes obtained for the glucose test were: 6.0; 6.0, and 7.0 and for starch test: 13.0, 16.0, and 11.0, respectively.

With regard to the above, a recommended sample size would be at least 12 study subjects/participants (16 due to possible study drop-outs or a lack of some single glucose results affecting the ability to calculate AUC).

Evaluation of the effect of the tested products on blood glucose concentration was conducted based on measurements and comparisons of glucose and insulin concentration at respective time points. The average of two independent concentration measurements (glucose, insulin) was determined for every subject at each time point. In order to refer obtained concentration values to the baseline level (at time point 0), the quotients T.X.0 were computed according to the following formula: (T.X/T.0) × 100, where T.X is a concentration level at time point X and T.0 is a concentration level at time point 0. Multiplication by 100 allows presentation of the results as a percentage change (with relation to the baseline level). The 0–240 min areas under the curves (AUCs) for the glucose/insulin concentrations were determined using the trapezoidal method. To determine whether the use of the IP/placebo on glucose/insulin concentrations was statistically significant, we used either the dependent-sample *t*-test or the Wilcoxon signed-rank test, depending on the distribution of the data, which was determined via the Shapiro-Wilk test. When the test was not significant and the data were normally distributed, we used the Wilcoxon signed-rank test. The reason for using the dependent-sample *t*-test is that the IPs and placebo were tested on the same group of subjects in each study. 

A significance level of alpha = 0.05 was used in all calculations. All statistical calculations were conducted with the use of the R software environment. Results are given as mean ± standard error (SE).

### 2.9. Ethical Approval and Consent to Participate

The studies were designed and conducted according to the Declaration of Helsinki and its amendments, to current GCP (Good Clinical Practice) guidelines, and to other appropriate local and international standards and guidelines concerning clinical trials. Moreover, study methodologies and endpoints were established based on EFSA (European Food Safety Authority) guidelines related to health claims concerning the effects of food products on postprandial blood glucose levels. The study protocols were approved by the local Human Research Ethics Committee (Medical University of Bialystok, Poland). All subjects signed informed consent forms prior to enrolment in the study.

## 3. Results

In Study 1, from a total of 36 screened subjects, 32 subjects were enrolled and divided into two groups with 16 subjects each. In Study 2, 180 subjects were screened, and 150 enrolled subjects were divided into 5 groups containing 30 subjects each. None of the subjects dropped off and the data of all enrolled subjects were analysed. The characteristics of the two study populations are presented in Table 1.

### 3.1. The Effect of IP-A/IP-B on Glucose and Insulin Concentrations after a High-GI/GL Meal (Study 1)

**Figure 2:**
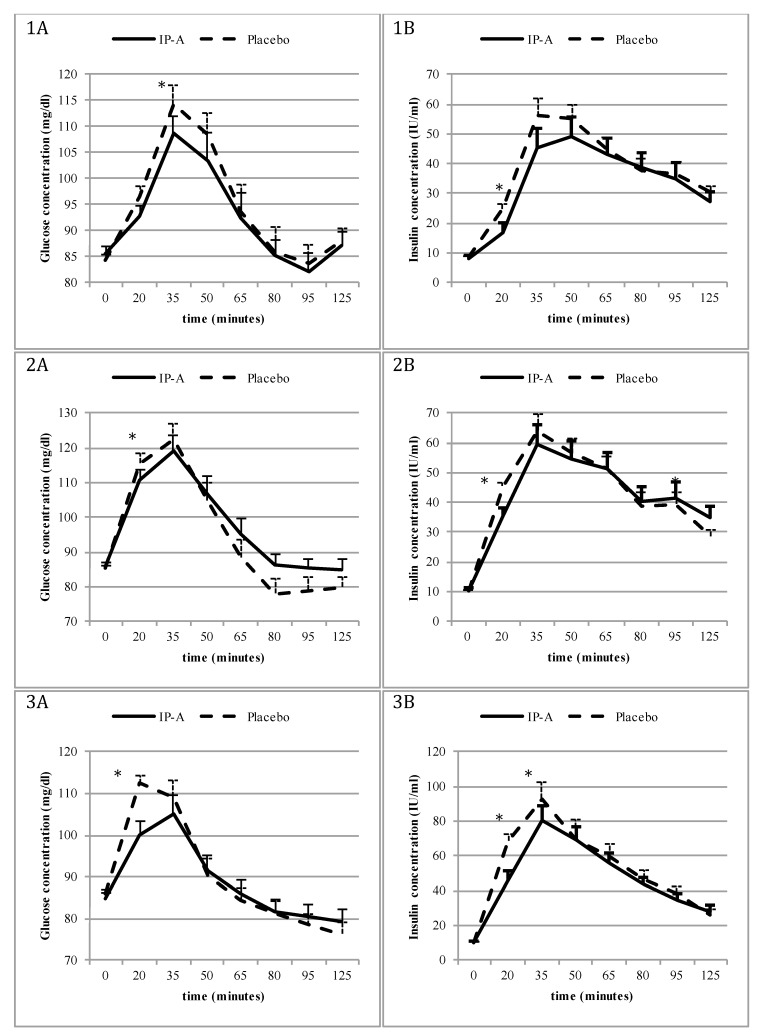

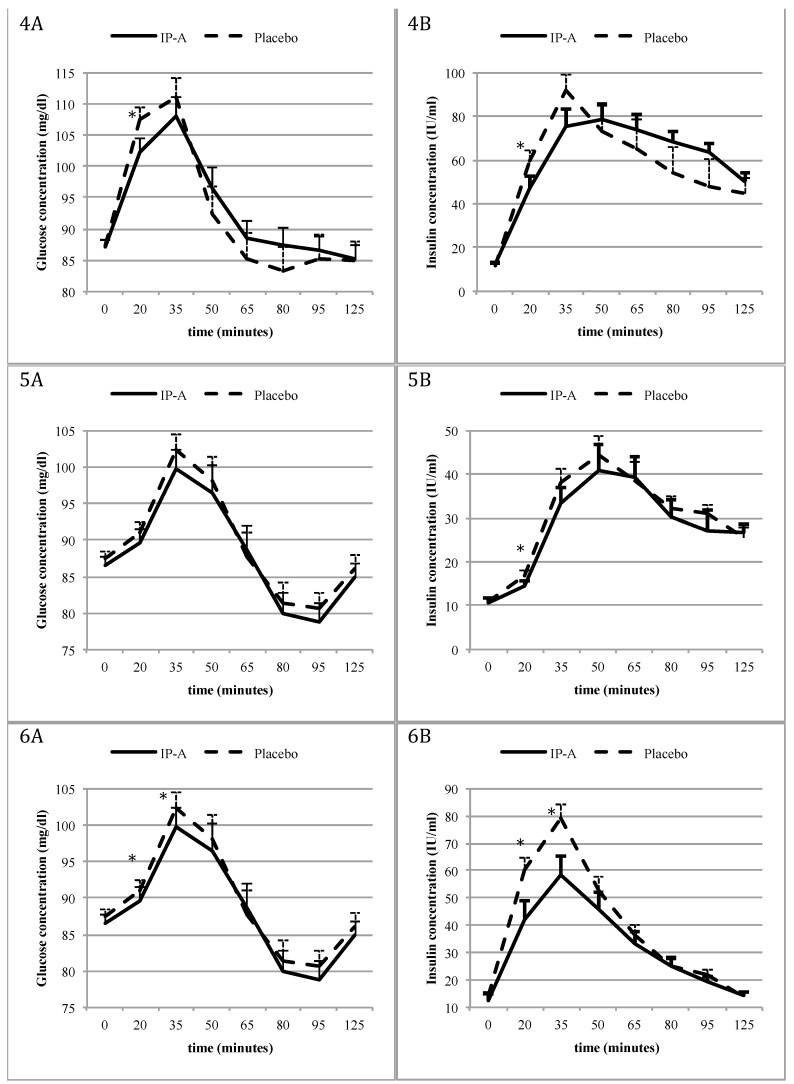
Fasting and postprandial (**A**) glucose (mg/dL) and (**B**) insulin (IU/mL) concentrations after investigational product-A (IP-A, solid lines) vs. placebo (dashed lines) administration during meal tests: 1. White roll (Study 1) 1A—glucose concentrations, 1B—insulin concentrations; 2. Cola and corn puffs (Study 2, Meal 1) 2A—glucose concentrations, 2B—insulin concentrations; 3. Breakfast cereal with milk (Study 2, Meal 2) 3A—glucose concentrations, 3B—insulin concentrations; 4. Muffin and yoghurt (Study 2, Meal 3) 4A—glucose concentrations, 4B—insulin concentrations; 5. Fries with ketchup (Study 2, Meal 4) 5A—glucose concentrations, 5B—insulin concentrations; 6. Pasta with cheese (Study 2, Meal 5) 6A—glucose concentrations, 6B—insulin concentrations. * *p* values < 0.05 were considered significant.

Lower blood glucose levels were observed following IP-A administration vs. placebo 20 min after intake of a white bread roll (92.8 ± 1.79 vs. 96.6 ± 2.09 mg/dL respectively, *p* = 0.07; Figure 2-1A); glucose levels became lower in the IP-A group compared to the placebo group 35 min after the meal intake (108.8 ± 3.64 vs. 114.3 ± 3.32 mg/dL, respectively, *p* = 0.039; Figure 2-1A). Insulin levels were also lower after IP-A administration than after placebo 20 min after meal intake (16.9 ± 1.55 vs. 24.9 ± 3.06 IU/mL, respectively, *p* = 0.003; Figure 2-1B); these levels showed a tendency to be lowered at 35 min compared to placebo, albeit not significantly (45.1 ± 5.73 vs. 56.1 ± 6.78 IU/mL, respectively, *p* = 0.07; Figure 2-1B). No differences were found between the IP-A/placebo groups in terms of the AUCs of the glucose and insulin concentrations over time; however, both the glucose and insulin curves flattened following IP-A administration compared to the placebo, with reduced maximum levels (peaks) of both parameters.

**Figure 3:**
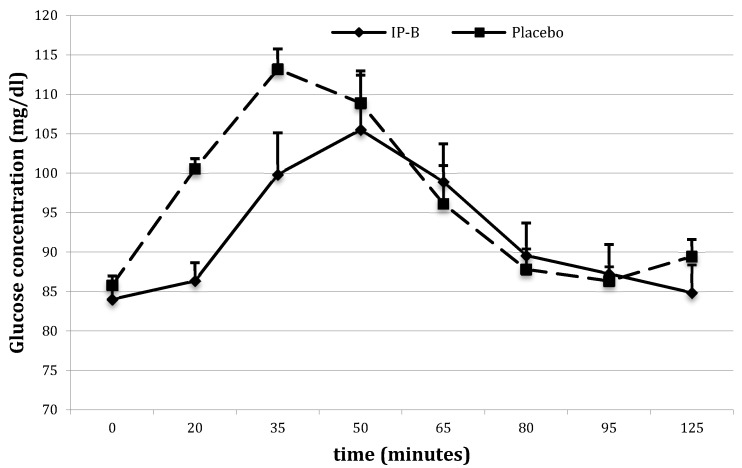
**Figure** 3. Average glucose concentration (mg/dL) after ingestion of the Investigational Product (IP) B vs. the placebo.

**Figure 4:**
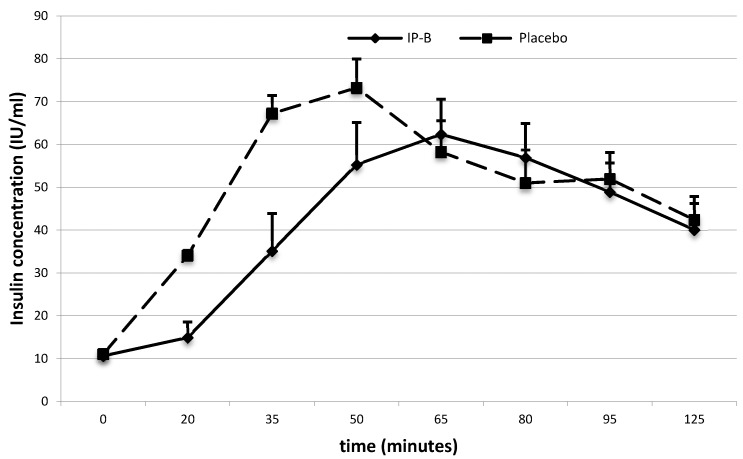
Average insulin concentration (IU/mL) after ingestion of the Investigational Product (IP) B vs. the placebo.

Lower blood glucose and insulin levels were also observed after administration of IP-B. Twenty minutes after meal intake, glucose levels were 86.3 ± 1.28 mg/dL after IP-B vs. 100.6 ± 2.29 mg/dL after the placebo (*p* < 0.01) (Figure 3), and insulin levels were 14.9 ± 1.22 IU/mL after IP-B vs. 34.1 ± 3.66 IU/mL after the placebo (*p* < 0.01) (Figure 4). The AUCs for the insulin concentration over time were 5213.4 ± 575.57 after IP-B administration and 6258.7 ± 747.79 after the placebo (*p* = 0.02).

### 3.2. Effect of IP-A on Glucose and Insulin Levels Following Various High-GI/GL Meals (Study 2)

Following Meal 1 intake (cola and corn puffs), lower blood glucose levels were observed after IP-A administration compared to the placebo at 20 min (110.6 ± 2.68 vs. 115.6 ± 2.99 mg/dL, respectively, *p* = 0.04; Figure 2-2A). However, glucose levels were similar after IP-A and placebo administration at 65 min (95.4 ± 5.08 and 88.6 ± 4.32 mg/dL, respectively, *p* = 0.09) and 95 min (85.6 ± 3.62 and 79.0 ± 2.54 mg/dL, respectively, *p* = 0.06). Lower insulin was observed after IP-A administration compared to the placebo 20 min after the meal intake (35.2 ± 4.74 vs. 45.3 ± 4.66 IU/mL, respectively, *p* < 0.01, Figure 2-2B). However, at 125 min, insulin levels in the IP-A group were higher than those in the placebo group (35.0 ± 3.58 vs. 28.7 ± 2.88 IU/mL, respectively, *p* = 0.01, Figure 2-2B). No differences were found for the remaining time points or for the AUCs of the glucose and insulin concentrations levels over time.

Following Meal 2 intake (breakfast cereal with milk), lower blood glucose concentrations were observed after IP-A administration compared to the placebo at 20 min (100.2 ± 1.97 vs. 112.5 ± 3.12 mg/dL, respectively, *p* < 0.01; Figure 2-3A); at the same time point, lower insulin concentrations were also noted after IP-A administration compared to the placebo (45.9 ± 4.02 IU/mL vs. 68.2 ± 5.53 IU/mL, respectively, *p* < 0.01; Figure 2-3B). No differences were found for the remaining time points or for the AUCs of the glucose concentrations over time, but the AUC for the insulin concentrations was significantly lower after IP-A intake compared to the placebo (5836.5 ± 585.35 vs. 6578.4 ± 454.78, respectively, *p* < 0.04).

Following Meal 3 intake (muffin and yoghurt), lower blood glucose concentrations were observed after IP-A administration compared to the placebo at 20 min (102.2 ± 1.9 vs. 107.5 ± 2.2 mg/dL, respectively, *p* = 0.005; Figure 2-4A); at the same time point, lower insulin concentrations were also noted after IP-A administration compared to the placebo (47.3 ± 4.7 vs. 59.9 ± 5.3 IU/mL, respectively, *p* = 0.01; Figure 2-4B). This trend was maintained for 35 min after meal intake (75.5 ± 7.3 vs. 92.1 ± 7.5 IU/mL, respectively, *p* = 0.069). No significant differences were found for the remaining time points or for the AUCs of the glucose and insulin concentrations over time.

Following Meal 4 intake (fries with ketchup), no statistically significant differences in blood glucose concentrations were observed during the entire postprandial period (Figure 2-5A), but insulin concentrations were lower after IP-A administration compared to the placebo 20 min after the meal intake (14.5 ± 1.24 vs. 16.8 ± 1.33 IU/mL, respectively, *p* = 0.02; Figure 2-5B). This trend was maintained for 35 min after meal intake (33.5.5 ± 2.92 vs. 90.4 ± 3.58 IU/mL, respectively, *p* = 0.07). No significant differences were found for the remaining time points or for the AUCs of the glucose and insulin concentrations over time.

Following Meal 5 intake (pasta with cheese), lower blood glucose concentrations were observed after IP-A administration compared to the placebo at 20 min (102.5 ± 1.99 vs. 110.3 ± 2.36 mg/dL, respectively, *p* < 0.01) and at 35 min (110.4 ± 3.10 vs. 116.2 ± 3.03 mg/dL, respectively, *p* = 0.04; Figure 2-6A). Insulin concentrations were also lower after IP-A administration compared to the placebo 20 min (41.8 ± 3.90 vs. 60.5 ± 6.98 IU/mL, respectively, *p* < 0.01; Figure 2-6B) and 35 min (58.2 ± 4.83 vs. 79.4 ± 7.37 IU/mL, respectively, *p* < 0.01) after meal intake. No differences were found for the remaining time points or for the AUCs of the glucose concentrations over time, but the AUCs of the insulin concentrations over time were lower after IP-A administration compared to the placebo (3938.1 ± 285.14 vs. 4802.8 ± 379.96, respectively, *p* = 0.01).

### 3.3. Effect of IP-A/IP-B on the Number of Hypoglycaemic Episodes

In Study 1, no hypoglycaemic episodes were observed. In Study 2, after Meal 1, five episodes of hypoglycaemia (<60 mg/dL) were observed after IP-A administration and 15 episodes were observed after placebo intake (*p* = 0.037). The minimum glucose concentrations after IP-A administration vs. placebo intake were 62.5 mg/dL vs. 49.5 mg/dL at 65 min, 51.0 vs. 33.0 mg/dL at 80 min, 47.5 vs. 44.5 mg/dL at 95 min, and 48.0 vs. 35.5 mg/dL at 125 min. At 65 min after Meal 5 intake, two episodes of hypoglycemia were reported following IP-A administration compared to eight after placebo intake (*p* = 0.105).

### 3.4. Safety of IP-A

In Study 1, five adverse events (AEs) were reported; most were probably related to the IPs and were resolved within several hours after IP intake. The reported AEs included gastrointestinal complaints of mild intensity (four subjects) and headache (one subject) of moderate intensity. No AEs related to IP-A were observed during Study 2.

## 4. Discussion

These studies allowed us to develop a new product (IP-A) named Tribitor ^®^ (MarMar Investment Sp. z o.o., Warsaw, Poland; Patent Application No. PCT/IB2015/052650 “Dietary compositions for reducing blood glucose levels and for weight management”) with an ability to decrease glucose and insulin peaks after intake of high-GI and high-GL meals. Tribitor^®^ (MarMar Investment Sp. z o.o., Warsaw, Poland) contains three different active ingredients: white mulberry extract (600 mg), white bean extract (1200 mg), and green coffee extract (400 mg), which may inhibit digestion and reduce carbohydrate absorption. Doses are based on the pilot study results (data on file). Overall, our studies indicate that Tribitor^®^ (MarMar Investment Sp. z o.o., Warsaw, Poland) can help control postprandial hyperglycaemia and has fewer side effects than the antidiabetic drug acarbose.

The gastrointestinal side effects of acarbose are caused by excessive inhibition of pancreatic α-amylase, resulting in abnormal bacterial fermentation of undigested carbohydrates in the large intestine. Alternatively, plant-based foods may have lower inhibitory activity against α-amylase but have stronger inhibitory activity against α-glucosidase [16]. Indeed, Adisakwattana et al. [17] showed that mulberry extract had high inhibitory activity against intestinal α-glucosidase but no inhibitory activity on pancreatic α-amylase. In another study, Banu et al. [18] gave 20 patients with T2DM plain tea (control group) and 28 patients with T2DM mulberry tea (test group). Fasting blood glucose concentrations were 153.5 ± 48.10 mg/dL in the test group and 178.6 ± 35.61 mg/dL in the control group [18]. After the consumption of plain or mulberry tea in addition to one teaspoon of sugar, there was a change in the postprandial blood glucose values between the two groups (i.e., 210.2 ± 58.73 mg/dL in the test group and 287.2 ± 56.37 mg/dL in the control group; *p* < 0.001) [18]. These results indicate beneficial effects of mulberry extract on the control of postprandial hyperglycaemia.

White bean extract has also been shown to affect postprandial glucose concentrations. A six-arm crossover study involving 13 randomized subjects (BMI 18–25) examined whether the addition of white bean extract would lower the GI of a commercially available high-GI food (white bread) [19]. A powder formula containing 1500 mg or 2000 mg of white bean extract caused insignificant reductions in the GI of white bread, while the 3000 mg dose caused a significant reduction in postprandial glucose concentrations (a reduction of 34.1%, *p* = 0.023) [19]. Tribitor^®^ (MarMar Investment Sp. z o.o., Warsaw, Poland) only contains 1200 mg of white kidney bean extract; however, the additive effects of the extracts used in the preparation allow a much lower dose to be used.

Regarding the addition of the green coffee extract used in Tribitor^®^ (MarMar Investment Sp. z o.o., Warsaw, Poland), Blum et al. [15] previously showed a significant decrease (*p* < 0.05) in post-load glycaemia (using an oral glucose tolerance test) after supplementation of 400 mg of green coffee in 15 healthy women and men, compared to the results obtained before supplementation (i.e., 133 ± 8.7 mg/dL after supplementation vs. 147.8 ± 9.3 mg/dl before supplementation). At the end of the study, an average weight loss of three pounds was noted [15]. In addition, coffee compounds such as chlorogenic acids can enhance glucose tolerance: in vitro and in vivo studies show that 5-caffeoylquinic acid can modulate glucose metabolism [20].

Tribitor^®^ (MarMar Investment Sp. z o.o., Warsaw, Poland) met all of the study endpoints. After IP-A administration, we observed lower postprandial glucose and insulin peaks, especially 20 and 35 min after the high-GI/GL meal intake. These positive effects were observed even though the studies were conducted on healthy people, without metabolic syndrome, pre-diabetes or T2DM, and with normal insulin secretion. In comparison, metformin (the first-line treatment for T2DM) did not significantly change the postprandial blood glucose concentrations in healthy subjects after single (850 mg, 1700 mg, or 2550 mg) or multiple (850 mg) doses, probably because the main mechanism of metformin action is improving insulin sensitivity, which in healthy people remains normal [21]. Therefore, the use of metformin for people without T2DM would be unreasonable, and Tribitor^®^ (MarMar Investment Sp. z o.o., Warsaw, Poland) may offer a suitable alternative to control postprandial hyperglycaemia.

There are multiple benefits of controlling postprandial glucose concentrations after the intake of high-GI/GL meals. Acute hyperglycaemia can adversely affect endothelial function, arterial walls, and the coagulation cascade, through mechanisms including oxidative stress. Thus, setting appropriate postprandial targets for blood glucose peaks is important for reducing the risk of arterial and microvascular disease [22,23,24]. Moreover, since hyperglycaemia and glucose toxicity affect β-cell secretion [25], lower postprandial glucose concentrations may have a protective effect on pancreatic β-cells. In our studies, we observed beneficial effects on postprandial glucose concentrations after all of the investigated high-GI/GL meals except fries with ketchup. In the case of fries with ketchup, it is probably the high fat content (30 g) of the meal that reduced the glycaemic response by itself, as shown previously [26]. However, as diets with a high fat content are known to lead to obesity, insulin resistance, T2DM, cardiovascular disease, and other serious disorders [27], this is not the right way to decrease the GI of the diet.

Despite the lack of differences in glucose concentrations after the consumption of fries, we still noted lower postprandial insulin concentrations after Tribitor^®^ (MarMar Investment Sp. z o.o., Warsaw, Poland) administration, which indicates less insulin was required to maintain the same blood glucose concentrations. Insulin resistance is associated with continuous exposure to high concentrations of insulin. Regardless of whether the insulin resistance or basal hyperinsulinemia came first, hyperinsulinemia itself might perpetuate insulin resistance [28]. We observed lower insulin concentrations after all of the investigated high-GI/GL meals following Tribitor^®^ (MarMar Investment Sp. z o.o., Warsaw, Poland) administration. This reduction in postprandial insulin concentrations is another positive effect of Tribitor^®^ (MarMar Investment Sp. z o.o., Warsaw, Poland) because hyperinsulinemia leads to insulin resistance [29] and its metabolic consequences [30]. In particular, postprandial hyperinsulinemia is independently associated with coronary artery disease, irrespective of fasting and postprandial glucose, and fasting insulin concentrations [31].

Along with the lower insulin concentrations observed after Tribitor^®^ (MarMar Investment Sp. z o.o., Warsaw, Poland) intake, we also noted fewer episodes of postprandial hypoglycaemia. Postprandial reactive hypoglycaemia appears within a few hours after high-GI meal consumption, when blood glucose concentrations begin to decline rapidly, primarily due to an exaggerated increase in insulin secretion, thereby resulting in hunger [32]. The observed trend towards lower glucose concentrations after placebo intake and cola and corn puffs consumption was probably due to a higher insulin response, which resulted in 3–4 times more episodes of postprandial hypoglycaemia compared to glucose concentrations after Tribitor^®^ (MarMar Investment Sp. z o.o., Warsaw, Poland) administration. Because one of the symptoms of hypoglycaemia is a sudden feeling of hunger, reducing the number of hypoglycaemic episodes can help limit uncontrolled eating between main meals, thereby helping to reduce or maintain body weight.

Overall, our studies show that this particular combination of plant extracts has synergistic effects on postprandial glucose metabolism: the effects of Tribitor^®^ (MarMar Investment Sp. z o.o., Warsaw, Poland) were more significant than the effects of administering the singular ingredients, even at higher doses. Thanks to the synergistic effects of the plant extracts—and the possibility of using lower doses of ingredients—we could minimize eventual side effects, as well as the production costs and the final price of the product.

The safety analysis showed that Tribitor^®^ (MarMar Investment Sp. z o.o., Warsaw, Poland) is safe in the single administration mode. Even if all the reported AEs (including gastrointestinal complaints) were probably related to Tribitor^®^ (MarMar Investment Sp. z o.o., Warsaw, Poland) they were of mild intensity and were resolved within several hours after IP intake. Our safety results are consistent with the findings of previous studies, which showed that the singular ingredients used in Tribitor^®^ (MarMar Investment Sp. z o.o., Warsaw, Poland) do not have any serious side effects and that the gastrointestinal side effects are rare and diminish upon extended use of the products [33,34]. Taking into account the issues of cost and the potential side effects of other weight-loss drugs available, Tribitor^®^ (MarMar Investment Sp. z o.o., Warsaw, Poland) should fulfil the market requirements despite the side effects mentioned above.

## 5. Conclusions

Our studies show the newly developed product Tribitor^®^ (MarMar Investment Sp. z o.o., Warsaw, Poland) can limit the disadvantageous consequences of consuming high-GI/high-GL meals. These data suggest that α-amylase and α-glucosidase inhibitors present in the white bean and mulberry extracts might be effective in decreasing the absorption of glucose from a carbohydrate-containing meal, and this activity is even more prominent when combined with a glucose transport inhibitor like green coffee extract. This can be especially helpful for people who have problems adhering to dietary restrictions. The long-term efficacy and safety outcomes of Tribitor^®^ (MarMar Investment Sp. z o.o., Warsaw, Poland), as well as its efficacy and safety in people with impaired glucose metabolism and T2DM, must be confirmed in further studies. However, our experiments suggest that Tribitor^®^ (MarMar Investment Sp. z o.o., Warsaw, Poland) can be used as an effective dietary supplement for the prevention and treatment of overweight, obesity, glucose intolerance, prediabetes, and T2DM, together with other dietary or medical recommendations.

## 6. Patents

Patent Application No. PCT/IB2015/052650.

## Figures and Tables

**Table 1:** Characteristics of the two study populations.

	Total (N)	Male/Female (*n*)	Age (years)	BMI (kg/m^2^)
Study 1	32	19/13	26.7 ± 3.7	26.51 ± 1.6
IP-A	16	10/6	26.9 ± 3.6	26.4 ± 3.8
IP-B	16	9/7	26.4 ± 1.6	26.7 ± 1.7
Study 2 (IP-A)	150	67/83	28.2 ± 9.2	25.5 ± 3.0
Meal 1	30	18/17	28.8 ± 9.6	25.6 ± 5.0
Meal 2	30	12/18	26.3 ± 7.8	25.6 ± 2.3
Meal 3	30	13/17	26.7 ± 8.2	25.2 ± 2.0
Meal 4	30	13/17	29.2 ± 9.9	25.6 ± 2.3
Meal 5	30	11/19	30.0 ± 9.8	25.6 ± 2.0

BMI: body mass index.

**Table 2:** The nutritional information of the tested meals.

Meal		Portion (g)	Energy (kcal)	Total Carbohydrate (g)	Total Protein (g)	Total Fat (g)
Study 1	White roll	150	481	92.0	13.1	6.0
	Water	200	-	-	-	-
	Total		481	92.0	13.1	6.0
Study 2 (Meal 1)	Cola ^1^	400	168	42.4	-	-
	Corn puffs ^2^	50	179	40.0	3.5	0.5
	Total		347	82.4	3.5	0.5
Study 2 (Meal 2)	Corn flakes ^3^	50	188	37.0	4.5	1.5
	Milk ^4^	400	272	42.0	12.0	6.0
	Total		460	79	16.5	7.5
Study 2 (Meal 3)	Muffin ^5^	120	293	54.9	6.0	9.0
	Yogurt ^6^	300	219	36.0	8.7	4.8
	Total		512	90.9	14.7	13.8
Study 2 (Meal 4)	Fries ^7^	200	598	74.0	6.8	30.0
	Ketchup ^7^	20	26	6.0	0.4	0.0
	Total		624	80.0	7.2	30.0
Study 2 (Meal 5)	Macaroni & Cheese Dinner ^8^	58+ water	220	41.0	6.0	3.0
	Total		220	41.0	6.0	3.0

^1^ The Coca-Cola Company, US; ^2^ Flips, Grupa BGK, Poland; ^3^ Cheerios, Nestle, Switzerland; ^4^ Mleko Wypasione Waniliowe, Mlekovita, Poland; ^5^ Starbucks, Poland; ^6^ Activia, Poland, ^7^ McDonalds, Poland; ^8^ Macaroni & Cheese Dinner; Kraft, US.
